# The myodural bridges' existence in the sperm whale

**DOI:** 10.1371/journal.pone.0200260

**Published:** 2018-07-09

**Authors:** Pei Liu, Chan Li, Nan Zheng, Xiaoying Yuan, Yutong Zhou, Pu Chun, Yanyan Chi, Campbell Gilmore, Shengbo Yu, Hongjin Sui

**Affiliations:** 1 The First Affiliated Hospital of Dalian Medical University, Dalian, China; 2 Department of Anatomy, College of Basic Medicine, Dalian Medical University, Dalian, China; 3 Dalian Hoffen Preservation Institution, Dalian, China; Sanya Institute of Deep-sea Science and Engineering Chinese Academy of Sciences, CHINA

## Abstract

Recent studies have identified that the myodural bridge (MDB) is located between the suboccipital muscles and cervical dura mater in the posterior atlanto-occipital interspace within humans. The myodural bridge has been considered to have a significant role in physiological functions. However, there is little information about the myodural bridge in marine mammals; we conducted this study to investigate and examine the morphology of the myodural bridge in a sperm whale. We also aim to discuss the physiological functions of the myodural bridge. In this study, a 15.1-meter long sperm whale carcass was examined. Multiple methods were conducted to examine the bridges of the sperm whale which included dissection, P45 plastination and histological analysis. This study confirmed the existence of the myodural bridge in the sperm whale and shows there are two types of the bridge in the sperm whale: one type was the occipital-dural bridge (ODB), the other type was the MDB. A large venous plexus was found within the epidural space and this venous plexus is thought to contain a great amount of blood when in deep water and thus the movements of suboccipital muscles could be a unique power source that drives cerebrospinal fluid circulation.

## Introduction

The suboccipital region is one of the most complicated areas of the human body. As the connection of the head and neck, it plays important roles in head movement and in nervous impulse transmission of the brain and spinal cord. A bridge connecting the suboccipital musculature and cervical dura mater was found in the atlanto-occipital interspace by Kahn [1], and termed the “myodural bridge (MDB)” by Hack [2]. The MDB was first described as an anatomical bridge, which bridges the epidural space between the rectus capitis posterior minor (RCPmi) and the cervical dura mater. A few years later, the existence of the MDB had been observed between the cervical dura matter and multiple suboccipital muscles including the RCPmi, the rectus capitis posterior major (RCPma) and the obliquus capitis inferior (OCI) [3]. The existence of bridges between the nuchal ligament (NL) and the dura mater is controversial as researchers debate its source [4–8].

Recently, several researchers have focused on the MDB and the results suggest that the MDB is physiologically important rather than just acting as fixation points of the spinal dura mater. The MDB actively and passively anchors the cervical dura, which prevents the infolding of the dura mater whenever the subocciptal muscles hyperextend [9–15]. The MDB transmits the tensile forces of the suboccipital muscles' movements to the cervical dura and modulates the velocity as well as the quantity of the cerebrospinal fluid (CSF) circulation/movement. Therefore the MDB could be taken as a power source of the CSF circulation/movement [8, 15–18]. Malformation and injuries of the RCPmi cause functional disorder of the MDB which contributes to the pathogenesis of cervicogenic headache [19–24].

The fact that MDB exists universally in mammals has been reported only in a few studies so far. Our previous studies have proved the existence of the MDB in seven terrestrial mammal orders [25], one reptilian species (the siamensis crocodile) [26] and six finless porpoise (*Neophocaena phocaenoides*) [27]. The objective of this study is to examine the morphology of the MDB within other marine mammals and to discuss possible physiological functions from an evolutionary perspective.

## Material

A 15.1-meter long sperm whale was acquired opportunistically examined in this study from stranding with the permission of Chinese Authorities for Animal Protection. It died naturally in the beache of Nantong (Jiangsu province, China). The cadaver was permitted for scientific research under the approval of the Ethics Committee of Dalian Medical University. The dead body of the collected sperm whale was perfused with 15% formalin solution for subsequent experiments. There were two main ways to the process of perfusion: the first way was arterial perfusion through the common carotid arteries of the sperm whale. The second way was local injection, which was a supplementary perfusion when arterial perfusion was insufficient. The perfusion effect was evaluated by the filling degree of the sperm whale's skin. Additionally, during the procedure of perfusion, we applied local injection of 15% formalin wherever formalin leakage was observed all over the body. The total consumption of 15% formalin was about 10 tons. The formal experiment took place after 12 months’ formalin fixation.

## Methods

### Dissection of suboccipital region

We conducted a layer-by-layer dissection of the neck from the dorsal midline of the carcass to explore the morphology of the RCDmi. We simultaneously started another layer-by-layer dissection from the right side of the neck to the dorsal midline. Gradually removing the skin, the subcutaneous fascia and the posterior occipital muscles, until the capsule of the atlanto-occipital joint was exposed. Afterwards we opened the capsule with a fenestration method to observe the inner structures of the atlanto-occipital joint including the occipital condyles and the attached soft tissue. Inside of the articular cavity, we removed the attached soft tissue in sagittal plane to observe the connection between the suboccipital muscles and the cervical dura mater. We then removed the attaching soft tissue from the intercondylar fossa of the occiput to the first thoracic vertebra, exposing the atlanto-occipital interspace and neighboring sclerous tissue.

### P45 sheet plastination

The carcass of sperm whale was sliced in the sagittal plane for a P45 sheet plastination. Anatomical structures of the posterior occipital region and connections between the suboccipital muscles and the cervical dura mater were observed. The brief experimental procedure is described below (Sui 2006). 3 mm transverse slices of the perfused specimens of the head and neck were made from side to side with a high-speed band saw. All the slices were bleached in 5% dioxogen overnight and dehydrated for casting with Hoffen polyester P45 (Dalian Hoffen Bio-Technique Co. Ltd., Dalian, P. R. China). After casting, the forced impregnation was made in a vacuum box and the absolute pressure was slowly decreased to 20, 10, 5, and 0 mmHg. Last, the sheets were cured using a heated water bath and were placed upright in the water bath at 40°C for 3 d. After curing, the sheets were removed from the bath and cooled to room temperature. Slices were then protected for observation and photograph.

### Histology of hematoxylin and eosin staining (HE)

The perfused sperm whale was dissected along the median sagittal plane to isolate a unit that included the MDB with the RCDmi, the posterior atlanto-occipital membrane and the spinal dura mater. Tissue blocks with thickness of 12–15 um and were dissected for Hematoxylin and Eosin staning.

All sections were dehydrated with ethanol and xylene and infiltrated with melted paraffin wax. The sections were then embedded in the filled mold and shaped when it had cooled to room temperature. A rotary microtome was conducted to make 6-μm-thick sections; the glass microscope slides were prepared for rehydration and staining with hematoxylin and eosin.

## Results

### Anatomy of the suboccipital region

#### The musculus rectus capitis dorsalis minor (RCDmi)

**Fig 1:**
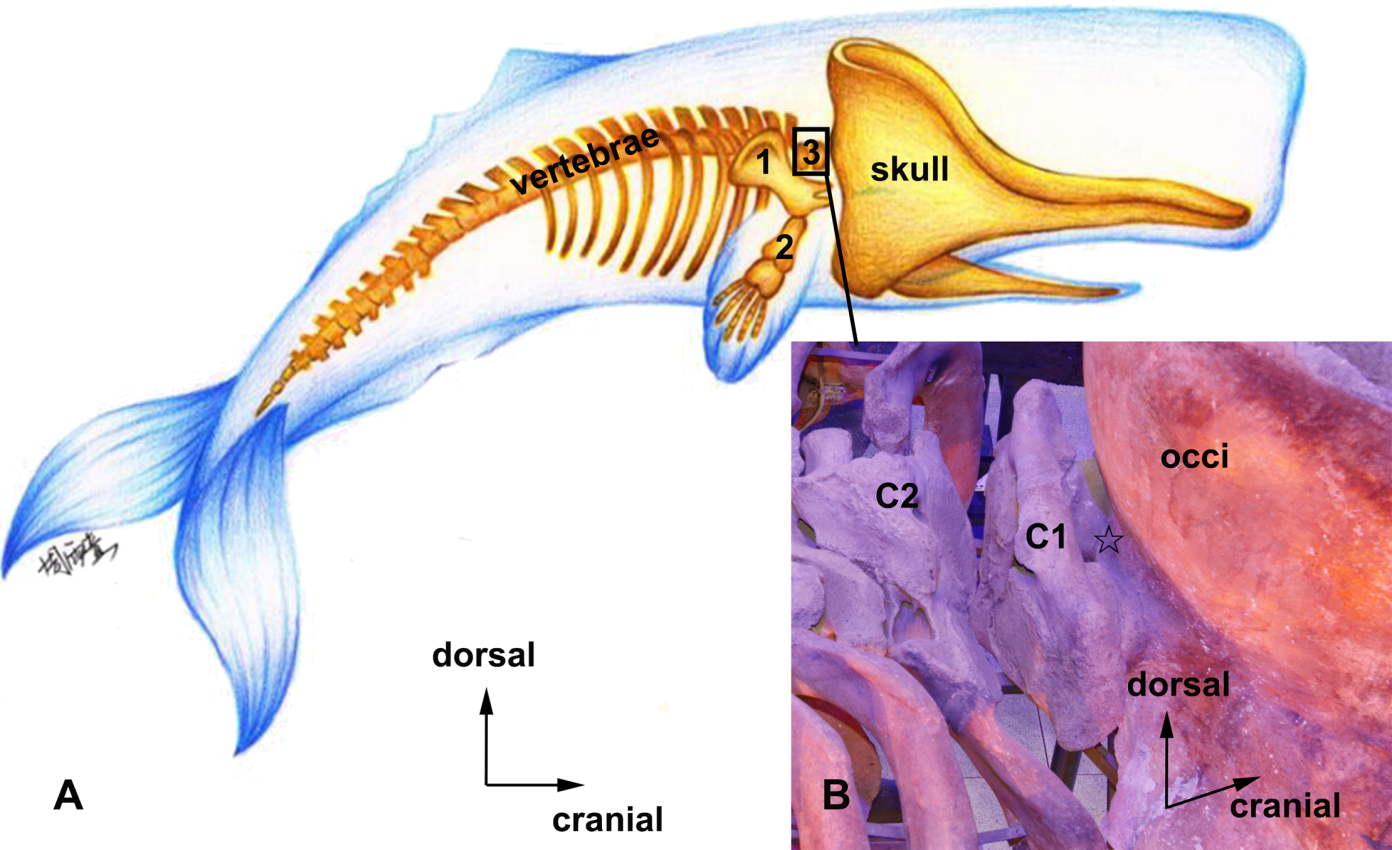
A basic sperm whale osteology model highlighting the enlarged skull and short cervical vertebrae (A). A photograph showing occiput (occi), atlas (C1), the fused vertebra (C2), and the atlanto-occipital interspace of the sperm whale (B). 1, scapula. 2, fin bone. 3, cervical vertebrae. occi, the occiput. C1, atlas. C2, the fused vertebra. ☆, the atlanto-occipital interspace.

**Fig 2:**
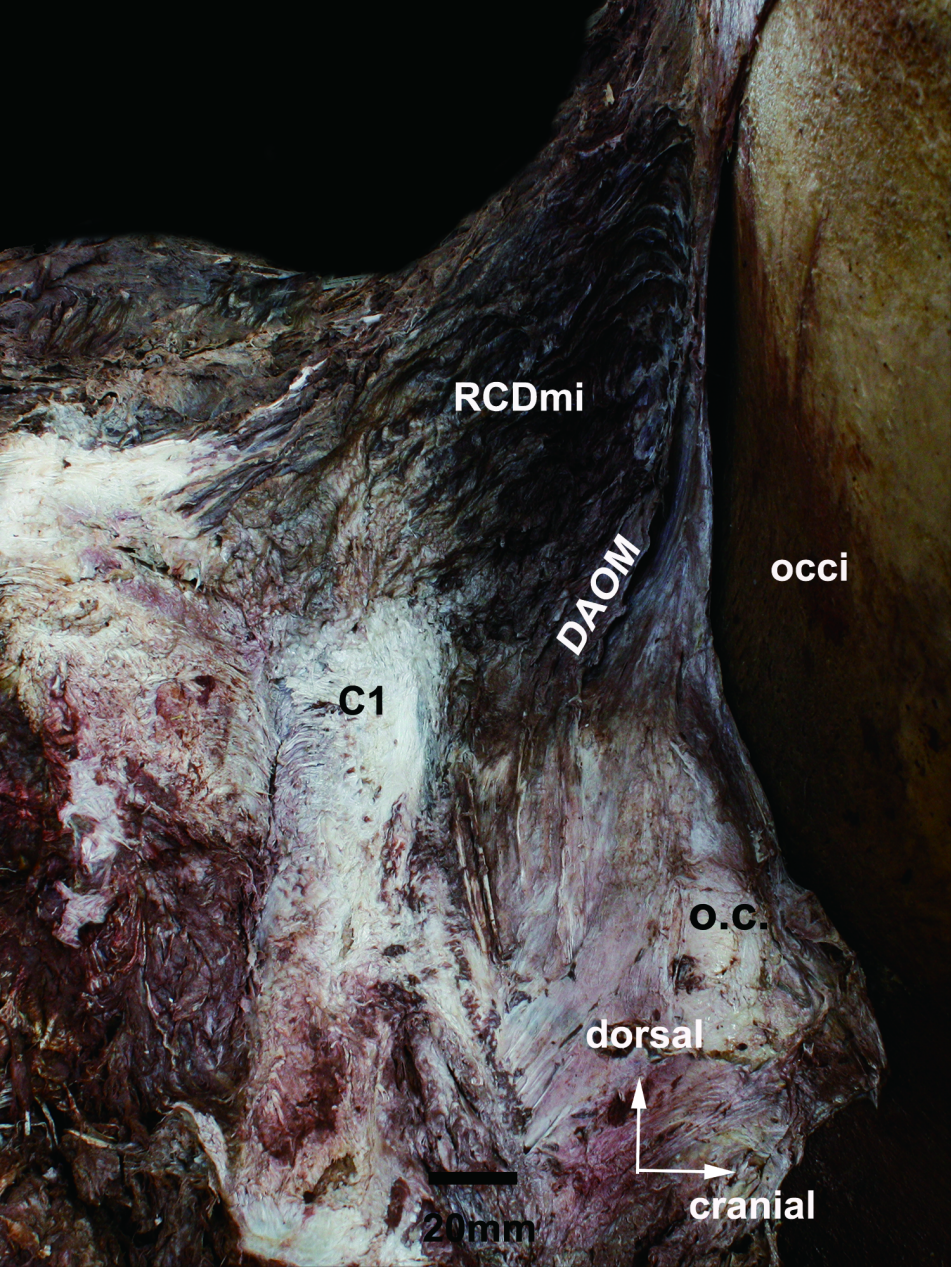
The posterolateral view of subocciptal musculature and connective tissue in the atlanto-occipital interspace showing occiput (occi), the musculus rectus capitis dorsalis minor (RCDmi), the dorsal atlanto-occipital membrane (DAOM), atlas (C1) and the the capsule of the atlanto-occipital joint (o.c.). occi, the occiput. RCDmi, the musculus rectus capitis dorsalis minor. DAOM, the dorsal atlanto-occipital membrane. C1, atlas. o.c., the capsule of the atlanto-occipital joint.

The sperm whale’s head is approximately 1/4 of its body length. The occiput and the occipital squama are positioned coronally and are almost perpendicular to the spine. The spinal cord was short and composed of two cervical vertebrae, including the atlas and the fused vertebra (C2) (Fig 1A). The upper boundary of the atlanto-occipital interspace was the caudal edge of the occipital squama; the lower boundary was the cranial edge of the atlas arch (Fig 1A). The atlanto-occipital interspace was enclosed by the dorsal atlanto-occipital membrane (DAOM), which originates from the upper boundary and terminated at the lower boundary of the atlanto-occipital interspace. The dorsal atlanto-occipital membrane continued with the capsule of the atlanto-occipital joint bilaterally (Fig 2).

According to the gross anatomy, the suboccipital muscles are located in dorsum of the cervical spine and in the lower occipital squama and the majority of the muscle bundles lie in the sagittal plane. The RCDmi originates from the dorsal part of the occipital squama and terminates at the dorsum of the atlas. The deep muscle bundles of the RCDmi covers the dorsal atlanto-occipital interspace above the dorsal atlanto-occipital membrane and a large amount of connective tissue was found amongst the muscle intervals (Fig 2).

#### The dorsal atlanto-occipital membrane (DAOM)

**Fig 3:**
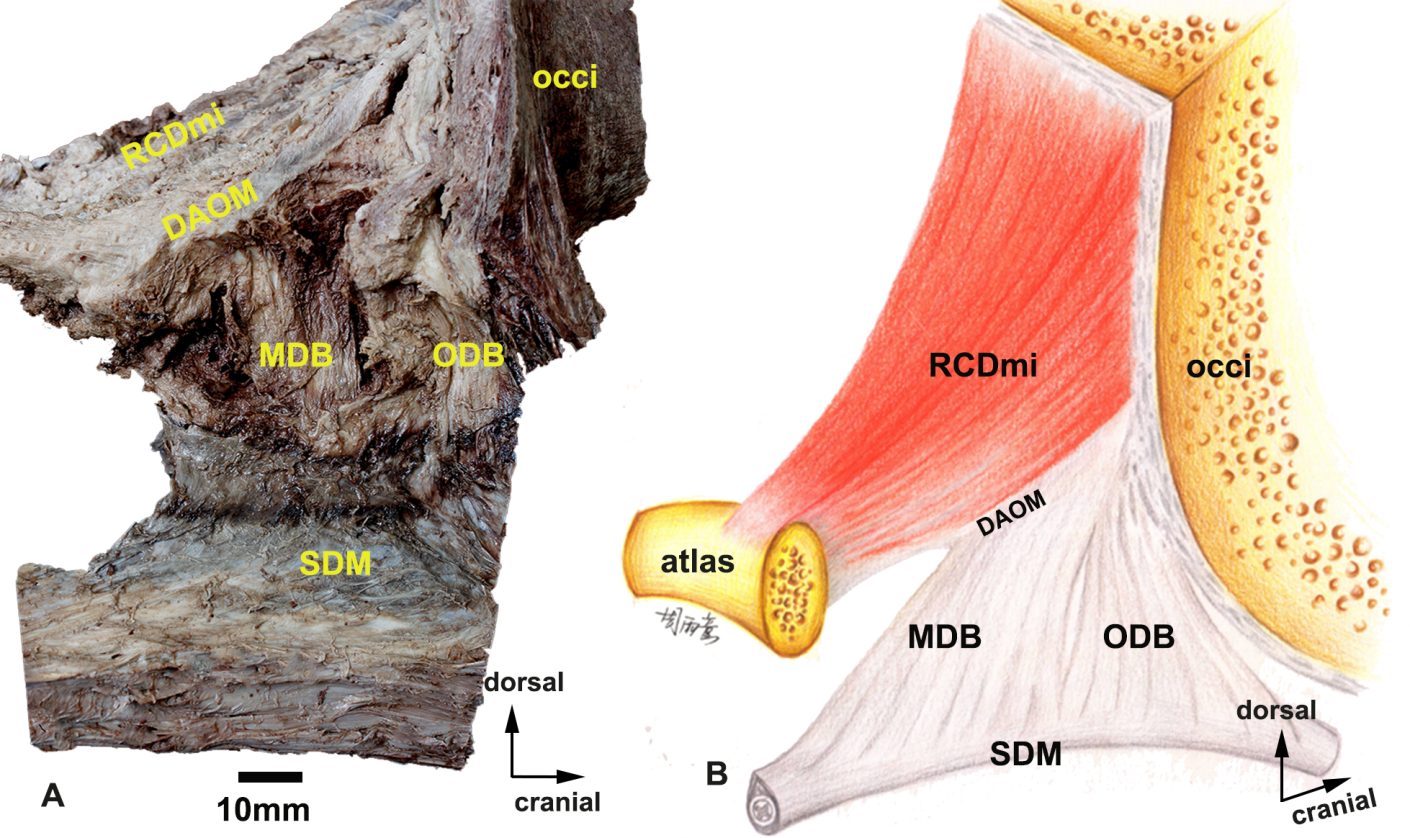
A photograph (A) and a model image (B) of the dissected connective bridges showing occiput (occi), the musculus rectus capitis dorsalis minor (RCDmi), the dorsal atlanto-occipital membrane (DAOM), the occipital dural bridge (ODB), the myodural bridge (MDB), and spinal dura mater (SDM). occi, the occiput. RCDmi, the musculus rectus capitis dorsalis minor. DAOM, the dorsal atlanto-occipital membrane. ODB, the occipital dural bridge. MDB, the myodural bridge. SDM, spinal dura mater.

Covered by the RCDmi, the dorsal atlanto-occipital membrane originates from the outer periosteal surface of the occiput and terminates at the dorsal arch of the atlas; it covers and closes the dorsal atlanto-occipital interspace. The middle part of the DAOM is thick and dense and both ends are thin and loose. The tissue of the DAOM runs bilaterally and continues with the capsule of the atlanto-occipital joint (Fig 3). The dorsal part of the DAOM fuse with the connective tissue in intervals of muscle bundles of the RCDmi.

#### The myodural bridge (MDB) and the occipital dural bridge (ODB)

To reveal the MDB, we dissected the cranial end of the DAOM and opened the atlanto-occipital interspace, which exposed the spinal canal. Along the median line, the DAOM gives off a dense bundle of fibers that form a sagittally-oriented sheet of fascia running ventrally and terminating at the dura mater, this is the MDB. For the ODB, we found that at the intercondylar fossa, the periosteal surface of the occiput is incrassated and in sagittal orientation with sheet-like fascia running ventrally and finally fusing with the spinal dura mater. The ODB is at the cranial end of the MDB in the atlanto-occipital interspace. We found that the epidural space of the sperm whale was predominantly broad and was densely filled with a venous plexus. (Fig 3)

### P45 plastination of the MDB and the ODB

**Fig 4:**
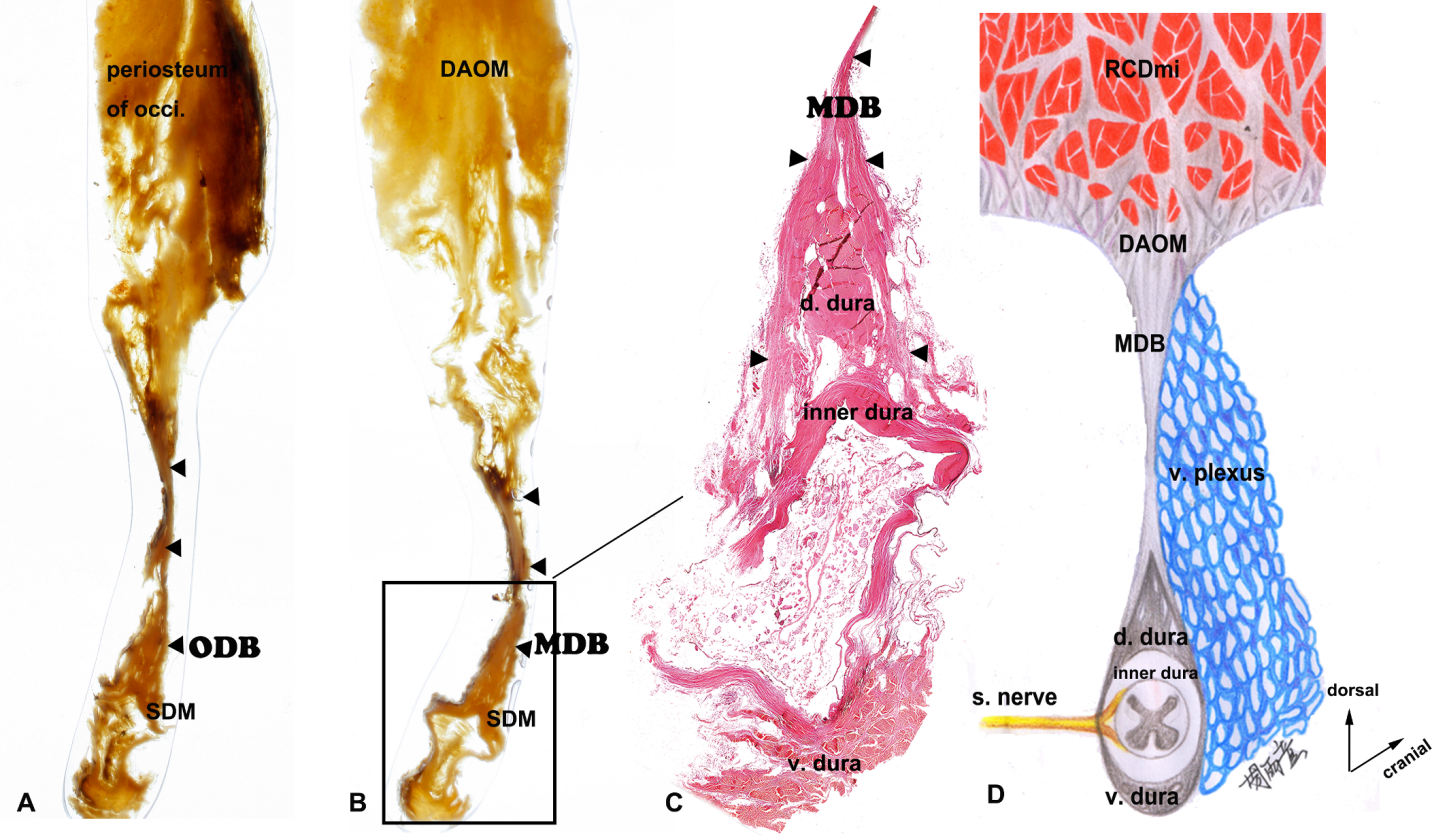
The transverse sectional view of the connective bridges and a model image. A: P45 sheet plastination slice of the the myodural bridge (MDB). B: P45 plastination slice of the the occipital dural bridge (ODB). C: Hematoxylin-Eosin slice of the the myodural bridge (MDB). D: a model highlighting the the musculus rectus capitis dorsalis minor (RCDmi), the dorsal atlanto-occipital membrane (DAOM), the myodural bridge (MDB), the venous plexus, the dura mater and the spinal nerve. periosteum of occi, periosteum of the occiput. RCDmi, the musculus rectus capitis dorsalis minor. DAOM, the dorsal atlanto-occipital membrane. ODB, the occipital dural bridge. MDB, the myodural bridge. SDM, spinal dura mater. d. dura, the dorsal dura. v. dura, the ventral dura. s. nerve, the spinal nerve. v. plexus, venous plexus. ▲, the fibers of the ODB and MDB.

Based on the observation of the P45 plastination sheets, the DAOM is composed of a large amount of randomly orientated fibers and gives off dense sheet-like fascia orientates ventrally which connects to the dura mater and aids in the formation of the MDB (Fig 4A, marked as “▲”). In addition, some of the sagittal fibers of the occipital periosteum compose the ODB; these fibers continue ventrally and form sheet-like fascia connecting to the dura mater (Fig 4B, marked as “▲”).

### The histology of the MDB and the SDM

According to the HE staining, the dura mater have two layers, the fibers of the inner layer circle around the spine and the outer layer shows its fibers orienting parallel with the spine. The fibers of the outer layer predominantly aggregate at the dorsal and ventral aspects of the dura mater and forms two funicular fibrous bundles along with the spine. Both of the lateral aspects of the dura mater are only covered by the fibers of the inner layer and the spinal nerves are found to break here. Dorsal to the dura mater, the fibers of the MDB (Fig 4C, marked as “▲”) divide into two funicular fiber bundles and wrap around the dorsal funicular bundle of the dura matter and finally fuse with the inner layer fibers of the dura mater. (Fig 4C and 4D)

## Discussion

Ever since the discovery of the human MDB in 1995 [2], studies from various perspectives have been done to verify the existence of and elucidate the physiological functions of the structure. In humans the MDB has been confirmed to exist between the deep suboccipital muscles, which includes the RCPmi [1, 2, 10, 28, 29], RCPma [13, 14, 29], OCI [12, 15, 29] and the cervical dura mater. The MDB has been found in the suboccipital region of quadrupedal terrestrial mammals, which includes cats, rats, dogs, rabbits, guinea pigs, macaques, and mandrill [25], as well as one reptilian species (the siamensis crocodile) [26]. Regarding marine mammals, we recently confirmed the existence of the MDB in the finless porpoise (*Neophocaena phocaenoides*) [27]. In this study, we confirmed the existence of the MDB in the sperm whale *via* multiple research methods. Unlike the terrestrial mammals, the sperm whale lives in the ocean and can dive to great depths. It features a very large head that accounts for 1/4~1/3 of the body length. Like many other whales and dolphins, the head of sperm whales has restricted movement because the cervical vertebrae (C2-C7) are fused [30]. According to our gross anatomy results there are two types of bridge both with different origins: the ODB which originates from the periosteal surface of the occiput and fuses with the dura mater. Since the periosteum of the occiput is tough and tenacious, the primary function of the ODB presumably is for fixation of the dura mater to stabilize the spinal cord. The other type of bridge (the MDB) originates from the RCDmi and may function to transmit the tensile forces from the RCDmi to the dura mater. It may also synergistically stabilize the dura mater as well as the spinal cord along with the ODB. The results of the P45 plastination and histological staining confirmed the functional speculation of the MDB the in sperm whale; the fibers of the MDB divided into two bundles and wrapped around the dorsal funicular fiber bundle of the dura mater and fused with both lateral parts of the cervical dura mater. Furthermore, the sperm whale only has the atlas and the fused vertebra (C2-C7) as its cervical vertebrae. The MDB only exists in the atlanto-occipital interspace; no MDB-like tissue was found in the atlanto-axial interspace. Collectively, these findings indicate that the sperm whale has a uniquely structured MDB and ODB when compared to terrestrial mammals. The periosteum of the occiput gives the anatomical origin of the ODB.

There are a variety of viewpoints concerning the function of the myodural bridge. Hack *et al*. [2] speculated that it protected the dura mater and spinal cord. They also stated that vertical fiber arrangement of the myodural bridge prevents infolding of the dura mater. Rutten *et al*. [10] proposed that the myodural bridge could maintain the flow of the CSF in the subarachnoid cavity and cerebellomedullary cistern during head movement. McPartland et al. [31] verified that the rectus capitis posterior minor muscle could prevent the infolding of the dura mater during head movement, which may inhibit CSF flow. In addition, McPartland *et al*. [31] and the study by Humphreys *et al*. [6] postulated that the MDB is implicated in cervicocephalic headaches; they found that the injured MDB could subsequently alter the volume of the subarachnoid space and attenuate the buffering of the cord impingement. Scali *et al*. [12, 15, 29] and Pontell *et al*. [13] also suggest that the myodural bridge is related to the maintenance of CSF outflow from the cisterna magna by maintaining the integrity of the subarachnoid space. Recently, Sui *et al*. [16] and Zheng *et al*. [8] proposed a new hypothesis addressing CSF circulation. They proposed that when the rectus capitis posterior minor contracted, the dura mater was pulled by the myodural bridge and thereby altering the subarachnoid volume and creating negative pressure. As a result, the CSF circulation could be affected by these changes; they advocated that this effect of the myodural bridge acts as an important source for CSF circulation. Xu *et al*. [17] investigated the CSF flow before and after head rotation using a phase-contrast cine magnetic resonance imaging method. They demonstrated that head rotation affected the mean velocity, flow rate and flow direction of CSF at the occipitocervical junction.

In humans, it is known that a venous plexus surrounds the cervical dura mater within the epidural space and in this study we found an extensive venous plexus within the sperm whale. Studies involving the use of sonar tracking and attached time-depth recorders found that the sperm whales could dive as deep as 1,000 meters. Consequeantly, sperm whales can experience 100 times the pressure that they do at surface. Whales usually have mass specific blood volumes that are three to four times those found in terrestrial mammals [32]. With the extreme crushing pressure, the venous plexus would be filled with great amount of blood and thus dramatically increase the pressure inside the spinal tube; this may significantly slow down the flow rate of the CSF circulation/movement [33–35]. Sperm whales also lower their heart rate when diving to depths which allows them to maintain moderate blood flow; however pulsations of the arteriesremain slow [32, 36–38]. When sperm whales swim at great depths, the MDB of these animals may contribute to transfering the tensile forces from the suboccipital muscles to the cervical dura mater and thereby continuously altering the volume of the subarachnoid space, acting as a unique mechanism to circulate the CSF. This is strong evidence to support our previous hypothesis that head movements have an important contribution to CSF circulation.

In this study, we have reported that there are two types of bridge-like tissue (MDB and ODB) existing between the RCDmi and the cervical dura mater within sperm whales. The sperm whale is one of the largest marine mammals, the existence of the MDB and ODB in this animal suggests that the anatomical bridge connecting the suboccipital muscles and the cervical dura mater may have been highly conserved during mammalian evolution and these findings collectively provide supportive information for studying the physiological function of human MDB.
